# Novel Quantitative Assay to Describe In Vitro Bovine Mastitis Bacterial Pathogen Inhibition by Non-*aureus* Staphylococci

**DOI:** 10.3390/pathogens11020264

**Published:** 2022-02-18

**Authors:** Bruno Toledo-Silva, Lisa Beuckelaere, Anneleen De Visscher, Chloë Geeroms, Evelyne Meyer, Sofie Piepers, Damien Thiry, Freddy Haesebrouck, Sarne De Vliegher

**Affiliations:** 1 M-Team and Mastitis and Milk Quality Research Unit, Department of Internal Medicine, Reproduction and Population Medicine, Faculty of Veterinary Medicine, Ghent University, 9820 Merelbeke, Belgium; lisa.beuckelaere@ugent.be (L.B.); chloe.geerooms@ugent.be (C.G.); sofie.piepers@ugent.be (S.P.); sarne.devliegher@ugent.be (S.D.V.); 2 Flanders Research Institute for Agriculture, Fisheries and Food (ILVO), Technology and Food Science, Agricultural Engineering, 9820 Merelbeke, Belgium; anneleen.devisscher@ilvo.vlaanderen.be; 3 Department of Veterinary and Biosciences, Faculty of Veterinary Medicine, Ghent University, 9820 Merelbeke, Belgium; evelyne.meyer@ugent.be; 4 Department of Infectious and Parasitic Diseases, Faculty of Veterinary Medicine and Institute for Fundamental and Applied Research in Animals and Health (FARAH), University of Liège, 4000 Liège, Belgium; damien.thiry@uliege.be; 5 Department of Pathobiology, Pharmacology and Zoological Medicine, Faculty of Veterinary Medicine, Ghent University, 9820 Merelbeke, Belgium; freddy.haesebrouck@ugent.be

**Keywords:** dairy cows, mastitis, growth inhibition, coagulase-negative staphylococci, *Staphylococcus aureus*, *Escherichia coli*, *Streptococcus uberis*

## Abstract

In this paper, we describe a new quantitative method to evaluate and quantify in vitro growth inhibition of mastitis-related bacteria. Colony-forming units of *Staphylococcus (S.) aureus* (*n* = 10), *Escherichia (E.) coli* (*n* = 10), and *Streptococcus (S.) uberis* (*n* = 10) were quantified after their growth on top of layers of trypticase soy agar (TSA) containing six different concentrations (varying from 10^2^ to 10^7^ CFU/mL) of bovine non-*aureus* staphylococci (NAS), i.e., *S. chromogenes* (*n* = 3) and *S. simulans* (*n* = 3) isolates. Growth inhibition of the mastitis-related major bacterial pathogens, including *E. coli*, was confirmed by all NAS, an effect that varied highly among NAS isolates and was not evident from the semiquantitative method with which the new method was compared. By subsequent application of the new method on a larger set of 14 bovine NAS isolates, we observed that *S. simulans* and NAS originating from teat apices (especially *S. epidermidis*) required lower concentrations to inhibit both methicillin-sensitive (MSSA) (*n* = 5) and methicillin-resistant *S. aureus* (MRSA) isolates (*n* = 5) originating from milk. Therefore, the new assay is a promising tool to precisely quantify the intra- and inter-species differences in growth inhibition between NAS.

## 1. Introduction

The predominant group of bacteria isolated nowadays from quarters with subclinical mastitis encompasses the non-*aureus* staphylococci (NAS) [1,2,3,4]. Bovine NAS form a heterogeneous group of staphylococcal species that differ in ecology, epidemiology, and virulence [5,6,7,8]. While NAS can have a negative impact on udder health [9,10], they are typically considered to be minor pathogens [11], not impacting milk yield [12]. In that respect, NAS might provide protection against intramammary infection (IMI) with major mastitis bacterial pathogens [13,14]. Over the years, our research group has reported multiple findings substantiating, to some extent, the latter concept [15,16,17,18,19,20]. 

Antibiotic resistance poses a threat to human and animal health and also concerns the dairy industry. Antimicrobials are used in the context of mastitis on dairy herds [21], thus novel strategies to tackle an IMI away from antimicrobials are needed, especially for an IMI caused by methicillin-resistant *Staphylococcus aureus* (MRSA) [22]. It has been shown that certain bovine NAS can inhibit the in vitro growth of major mastitis bacterial pathogens, including *S. aureus*, *Streptococcus* (*S.*) *dysgalactiae, S. uberis* [15,23], and even *Escherichia* (*E*.) *coli* [24], opening venues for probiotic approaches for bovine mastitis prevention and control [25]. The method previously used [15,23,24] is a semiquantitative method that can be employed to screen a large group of isolates, relatively fast for their ability to inhibit the growth of major mastitis bacterial pathogens, especially those that are Gram positive, yet we hypothesize that a novel quantitative method would allow us to interpret (more) intra- and inter-species variations between NAS and also their effect against other bacterial strains. 

In that respect, we recently demonstrated that in vitro *S. aureus* growth inhibition by bovine NAS only plays a minor role in the regulation of the *S. aureus agr* related virulence factors [18] and was not important for *S. aureus* biofilm formation and dispersion [19]. However, the semiquantitative method used to evaluate the in vitro growth inhibition of *S. aureus* likely did not provide the necessary detail regarding the extension and variability of the inhibitory effects of the different bovine NAS to draw final conclusions. Therefore, we aimed in this paper (1) to describe a novel method that quantifies precisely whether NAS can inhibit the growth of major mastitis bacterial pathogens and (2) to compare the results with the previously described semiquantitative in vitro growth inhibition method; in addition, (3) the new method was applied on another set of well-described bovine NAS isolates in order to study growth inhibition of methicillin-sensitive (MSSA) and methicillin-resistant *S. aureus* (MRSA) isolates originating from milk.

## 2. Results

### 2.1. Novel Quantitative Growth Inhibition Assay

An average of 9.53 (0–10), 10, and 8.19 (5–10) log_10_ CFU/mL for *S. aureus*, *E. coli*, and *S. uberis* isolates, respectively, were obtained for analyses for each NAS isolate. 

#### 2.1.1. *Staphylococcus aureus*

For *S. chromogenes* TA, a concentration of 5.10 log_10_ CFU/mL was enough to stop the growth of *S. aureus*. *Staphylococcus chromogenes* IM and *S. simulans* EL38 were able to stop the growth of *S. aureus* if the NAS concentration was 6.13 or 6.19 log_10_ CFU/mL, respectively. A concentration of 8.24 log_10_ CFU/mL of *S. simulans* EL12 or *S. simulans* EL29 was sufficient to stop the growth of *S. aureus*, while a concentration of 8.34 log_10_ CFU/mL of *S. chromogenes* EL14 was needed in order to obtain the same result.

#### 2.1.2. *Escherichia coli*

*Staphylococcus chromogenes* IM and *S. chromogenes* TA stopped the growth of *E. coli* when a concentration of 6.13 or 6.15 log_10_ CFU/mL was used in the top layer. The growth of *E. coli* was not stopped by *S. simulans* EL38, *S. simulans* EL12 or *S. chromogenes* EL14 if the NAS concentration was lower than 8.19, 8.24 or 8.34 log_10_ CFU/mL, respectively, while a concentration of 9.24 log_10_ CFU/mL of *S. simulans* EL29 was needed to inhibit *E. coli* from growing.

#### 2.1.3. *Streptococcus uberis*

A concentration of 4.74 log_10_ CFU/mL of *S. simulans* EL38 was enough to stop the growth of *S. uberis*. *Staphylococcus chromogenes* IM, *S. chromogenes* TA, *S. simulans* EL12 and *S. simulans* EL29 were able to stop the growth of *S. uberis* when a concentration of 5.00, 5.15, 5.65, or 5.95 log_10_ CFU/mL was used in the top layer, while a concentration of 8.34 log_10_ CFU/mL of *S. chromogenes* EL14 was required before the growth of *S. uberis* was stopped.

*S. simulans* EL12, *S. chromogenes* TA, and *S. simulans* EL29 were able to stop the growth of *S. uberis* when a concentration of 5.00, 5.65, 5.70, or 5.95 log_10_ CFU/mL was used in the top layer, while a concentration of 8.34 log_10_ CFU/mL of *S. chromogenes* EL14 was required before the growth of *S. uberis* was stopped.

In total, 25 NAS plates of the 228 plates (11%) were contaminated on day 4, making CFU counting impossible. All plates with all the EL14 NAS concentrations in combination with *S. uberis* were contaminated, so the experiment was repeated for these 12 plates. For EL38, all *S. aureus* isolates of NAS concentration of 4.19 log_10_ CFU/mL were contaminated, so the data for this NAS concentration for *S. aureus* are missing. These contaminations have led to the loss of results for some of the major mastitis bacterial pathogen isolates.

### 2.2. Existing Semiquantitative Growth Inhibition Assay

The results of the semiquantitative in vitro growth inhibition method used to test the ability of each NAS isolate to inhibit the growth of *S. aureus, E. coli,* and *S. uberis* are shown in Table 1. The average size of the inhibition zone (mm) is shown for *S. aureus* (measured in duplicate), *E. coli* (measured in triplicate), and *S. uberis* (measured in triplicate), together with the type of inhibition on the central-streak zone.

#### 2.2.1. *Staphylococcus aureus*

Only for *S. chromogenes* TA, a zone of total growth inhibition (7 mm) of *S. aureus* could be detected, while the other NAS isolates were capable to inhibit the growth of *S. aureus* only partially. Still, small differences could be seen between *S. chromogenes* IM (12 mm) and the four other NAS isolates (15 mm). *Staphylococcus chromogenes* TA was classified as a total growth inhibitor, while the other NAS isolates were classified as isolates with the capability to partially inhibit the growth of *S. aureus*.

#### 2.2.2. *Escherichia coli*

None of the NAS isolates displayed a zone of total growth inhibition for *E. coli*, but for both *S. chromogenes* IM and *S. chromogenes* TA, a small zone (2 mm) of partial growth inhibition could be detected. All tested NAS isolates were classified as incapable of inhibiting the growth of *E. coli*.

#### 2.2.3. *Streptococcus uberis*

While there was no zone of total growth inhibition of *S. uberis* for any of the NAS isolates, differences could be detected between NAS isolates regarding size of the partial growth inhibition zone. *Staphylococcus chromogenes* IM, *S. chromogenes* TA, and *S. simulans* EL12 had a partial growth inhibition zone of 3 mm, while the partial growth inhibition zone was 2 mm for *S. simulans* EL29. *Staphylococcus chromogenes* EL14 and *S. simulans* EL38 had the smallest partial growth inhibition zone for S. uberis (1 mm). The classification of *S. chromogenes* IM, *S. chromogenes* TA, and *S. simulans* EL12 was as a partial growth inhibitor, while *S. chromogenes* EL14, *S. simulans* EL29, and *S. simulans* EL38 were classified as isolates incapable of inhibiting the growth of *S. uberis*.

### 2.3. Comparison between the Novel Quantitative Assay and the Existing Semiquantitative Growth-Inhibiting Assays

**Figure 1:**
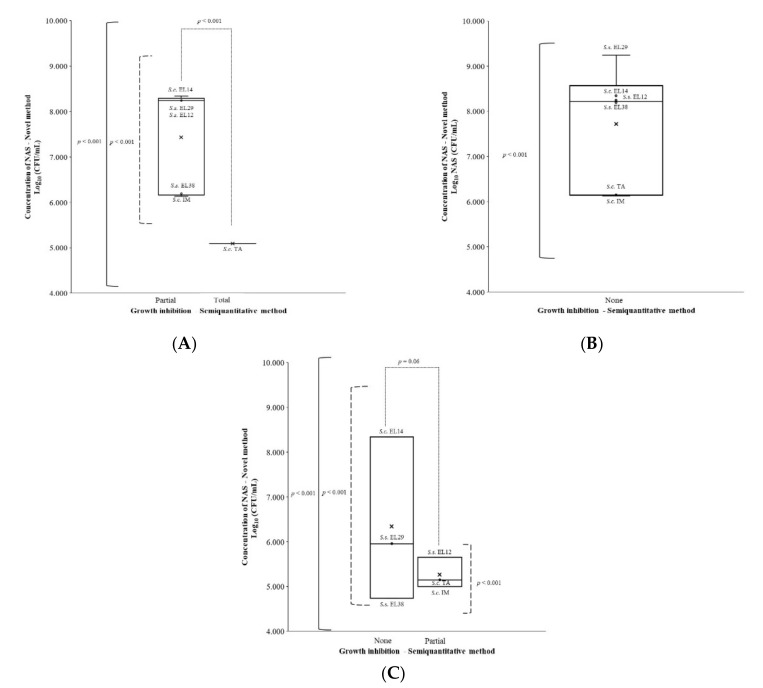
Non-*aureus staphylococcus* concentrations (Y-axis), as determined with the novel method needed to inhibit the growth of *Staphylococcus aureus* (**A**), *Escherichia coli* (**B**), and *Streptococcus uberis* (**C**) for each category (total, partial, or no growth inhibition) of the NAS isolates as determined by the previously described semiquantitative method [15] (X-axis). The average concentration (CFU/mL) of an NAS category as determined by the semiquantitative method (total, partial, or no growth inhibition) necessary to inhibit the growth of the major mastitis bacterial pathogen is represented by ‘x’. Solid lines represent statistical differences among all NAS isolates (S.c.: *S. chromogenes* and S.s.: *S. simulans*), whereas dotted lines represent differences between NAS isolates classified in different categories by the semiquantitative method (total, partial, or no growth inhibition) and dashed lines represent differences within NAS isolates classified in the same category.

The average NAS concentration at which the growth of *S. aureus*, *E. coli*, or *S. uberis* were inhibited at a function of the final classification of the NAS isolates, based on the semiquantitative in vitro growth inhibition method, is shown in Figure 1.

#### 2.3.1. *Staphylococcus aureus*

The concentration of NAS at which the growth of *S. aureus* was inhibited varied significantly among the different NAS isolates, independently of their capability to inhibit *S. aureus* growth via the semiquantitative method (*p* < 0.001) (Figure 1A). Moreover, the concentration at which *S. chromogenes* TA (5.10 log_10_ CFU/mL), classified as the only total inhibitor of *S. aureus*, inhibited *S. aureus*, was significantly lower compared to the concentration at which the NAS classified as partial inhibitor (based on the semiquantitative method) inhibited growth of *S. aureus* (7.39 log_10_ CFU/mL). Additionally, by using the novel method, a significant difference (*p* < 0.001) was observed in the growth-inhibitory effect among the different NAS isolates. Remarkably, all these NAS isolates were classified as partial inhibitors by the semiquantitative method. Furthermore, 2 log_10_ CFU/mL differences were observed between these NAS isolates, as is shown in Figure 1A. 

#### 2.3.2. *Escherichia coli*

According to the semiquantitative method, all isolates were classified as not being able to inhibit the growth of *E. coli*. Based on the novel method, an average concentration of 7.72 log_10_ CFU/mL was needed to inhibit the growth of *E. coli*. Still, among the different NAS isolates, there was significant variation (*p* < 0.001) in the concentration at which the growth of *E. coli* was inhibited (3 log_10_ CFU/mL; Figure 1B).

#### 2.3.3. *Streptococcus uberis*

Among the NAS strains, the concentration at which *S. uberis* stopped growing varied significantly, independent of their capability to inhibit *S. uberis* growth via the semiquantitative method (*p* < 0.001) (Figure 1C). The concentration at which *S. uberis* was inhibited from growing tended to be significantly higher for NAS classified as not being capable of inhibiting the growth of *S. uberis* (6.35 log_10_ CFU/mL) (based on the semiquantitative method) compared to NAS isolates being classified as partial inhibitors (5.41 log_10_ CFU/mL) (*p* = 0.06). In the group of NAS isolates classified as not being capable of inhibiting the growth of *S. uberis* (based on the semiquantitative method), almost 4 log_10_ CFU/mL differences could be observed between the different NAS isolates (*p* < 0.001), while almost 1 log_10_ CFU/mL differences could be observed between the partial inhibitors of *S. uberis* (*p* < 0.001).

### 2.4. Application of the Novel Quantitative Growth Inhibition Assay

**Figure 2:**
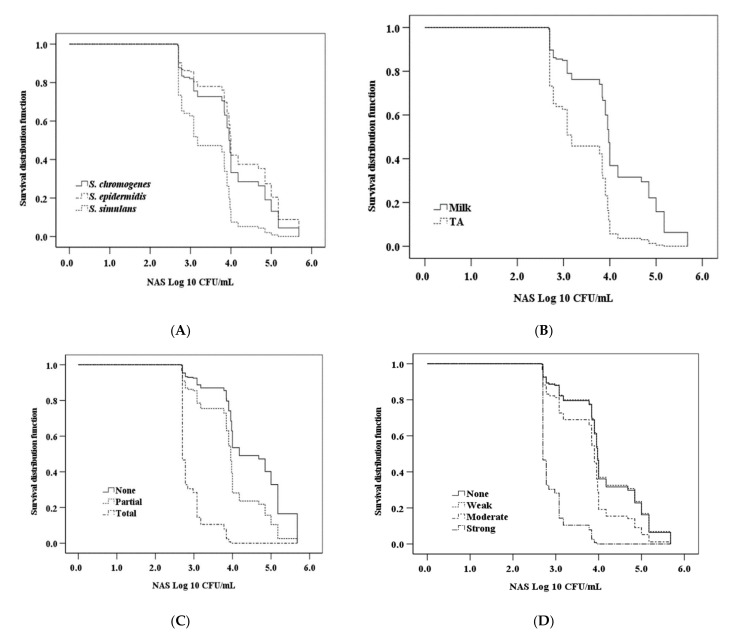
Kaplan–Meier survival curves of *Staphylococcus aureus* growth at different concentrations of bovine non-*aureus* staphylococci (NAS). For the novel assay, different *S. aureus* isolates (*n* = 10) were inoculated on top of plates, each containing a different concentration of *S. chromogenes, S. epidermidis*, and *S. simulans* isolates (**A**); isolates originating from milk or teat apices (TA) (**B**); isolates with the capacity to inhibit the growth of *S. aureus* according to a semiquantitative assay (or not) [18] (**C**); and isolates able to repress the *agr* system of *S. aureus* (or not) (**D**).

Overall, NAS as a group (comprising *S. chromogenes, S. epidermidis*, and *S. simulans*) inhibited the growth of all *S. aureus* isolates at an average concentration of 5.34 log_10_ CFU/mL (Figure 2). 

In the new assay, the NAS isolates classified as total inhibitors (hazard ratio (HR): 16.24; 95% confidence interval (CI): 6.93–38.01) by the semiquantitative method [18] as well as those NAS isolates classified as partial inhibitors (HR: 2.03; CI: 1.10–3.73) were significantly more likely to inhibit *S. aureus* growth than those NAS isolates that did not have any inhibitory effect (Figure 2A). 

*Staphylococcus simulans* was significantly more likely to inhibit the growth of S. aureus compared to *S. chromogenes* (HR: 2.35; CI: 1.50–3.68), whereas the difference between *S. chromogenes* and *S. epidermidis* isolates was not significant (HR: 0.78; CI: 0.50–1.22; *p* = 0.28; Figure 2B). Moreover, the growth-inhibitory effect of NAS isolates originating from TA was significantly more pronounced than that of NAS isolates originating from milk (HR: 2.88; CI: 1.87–4.41; Figure 2C). The NAS isolates that previously [18] demonstrated moderate (HR: 1.62; CI: 0.98–2.69, *p* = 0.06) and strong (HR: 9.88; CI: 4.94–19.75) capacity to repress the *agr* system of *S. aureus* were more likely to inhibit the growth of *S. aureus* than NAS isolates that had no such capacity (Figure 2D). 

Additionally, the NAS isolates equally inhibited the growth of MRSA and MSSA isolates (HR: 0.94; CI: 0.67–1.34).

The significant interaction between “species” and “origin” revealed that the inhibitory effect of TA isolates was more pronounced in *S. epidermidis*, than in *S. simulans* and *S. chromogenes*.

## 3. Discussion

We demonstrated that our novel quantitative in vitro growth inhibition method is consistent with the semiquantitative in vitro growth inhibition method yet adds much more detail on the extension of inhibition by NAS, showing that the inhibitory concentration strongly depends on the NAS isolates. Additionally, we demonstrated that this new method is applicable to screen the inhibitory effect of NAS on the growth of MRSA and MSSA isolates, an effect that was more pronounced in *S. simulans*, NAS originating from TA, and NAS with moderate or strong capacity to repress the *agr* system of *S. aureus*. Some *S. aureus* isolates seem to be more susceptible to the NAS effect than others, yet this susceptibility is not associated with their antimicrobial resistance. 

The quantitative method was able to detect the in vitro growth inhibition of *S. aureus, E. coli*, and *S. uberis* by NAS. As we expected, the inhibitory effect varied among the NAS isolates and the major mastitis bacterial pathogens. Yet, the concentration required to achieve the inhibitory effect of the same major pathogen appears to depend on the NAS isolate. For instance, the lowest concentration required to stop the growth of *S. aureus* was achieved by *S. chromogenes* TA (5.10 log_10_ CFU/mL), whereas it was achieved by *S. simulans* EL38 (4.74 log_10_ CFU/mL) for *S. uberis*. Strikingly, the new method was also shown to be highly effective in detecting growth inhibition of *E. coli*. Although higher concentrations were necessary to achieve such effect when compared to the other major pathogens (average concentration of 7.72 log_10_ CFU/mL), this finding supports our hypothesis that the growth-inhibitory effect strongly depends on the NAS. These inhibitory effects are most likely due to the capacity of bovine NAS to produce bacteriocins [26,27,28,29,30], in which NAS species and habitat are likely determinant in this process.

Previously, De Vliegher et al. [15] reported on differences in the intensity of inhibition of mastitis-related pathogens such as *S. aureus*, *S. dysgalactiae, S. uberis*, and *E. coli* by *S. chromogenes*. The authors observed pronounced inhibitory effects of *S. chromogenes* isolates against *S. aureus*, variable effects against *S. dysgalactiae* and *S. uberis*, and no effects against *E. coli*, suggesting that this susceptibility to NAS is more pronounced against phylogenetically-related bacterial species (i.e., Gram positive). Other studies also reported only a modest effect against Gram-negative bacteria; however, this effect was observed by using the semiquantitative growth inhibition assay [15,23,24,31]. On the other hand, bacterial growth can also be limited by the availability of nutrients, which differs according to the local environment. At any type of starvation, *E. coli* can pass into a dormant mode to protect their cells against harsh conditions [32]. The competition between NAS and *E. coli* for limiting nutrients over time during growth could at least partially explain our results and needs more study. Other Gram-positive bacteria originating from the bovine mammary gland, such as lactic acid bacteria (LAB), have also been reported to inhibit *E. coli* [33], (some) by the production of organic acids [34]; however, this antimicrobial effect is reported to be limited.

We demonstrated that our novel method is able to achieve results that reflect those of the semiquantitative method [15], although more and very important differentiation between NAS isolates were obtained. Furthermore, the method was applicable and suitable to identify similar growth inhibition of both MRSA and MSSA isolates by another subset of NAS isolates (selected from Toledo-Silva et al. [18,19]), in lower concentrations than in the semiquantitative method, as previously reported by our group [18]. Growth inhibition of MRSA by using auto-inducing peptides (AIP) produced by NAS has been reported [35]. Although the growth-inhibitory effect was related to some extent to the capacity of our NAS isolates to repress *agr*, we assume that this effect was probably co-founded by the previously reported effects of NAS species and habitats [18].

The emergence of MRSA isolates as the cause of mastitis in dairy cows has become a growing concern for public health [36]. In that respect, an intriguing finding is that MRSA were equally susceptible to NAS as MSSA. Previously, Carson et al. [30] reported the growth inhibition of human MRSA isolates by six bovine NAS species, including *S. chromogenes*, *S. simulans*, and *S. epidermidis* using the semiquantitative growth inhibition method, an effect that was explained by the capacity of NAS to produce bacteriocins. Revealing and quantifying the inhibition of MRSA by NAS is an important finding of our study and sheds new light on the search for alternative treatments for bovine mastitis caused by these resistant pathogens.

In the semiquantitative growth inhibition method, one isolate of *S. simulans* and one isolate of *S. chromogenes*, both originating from TA, totally inhibited the growth of *S. aureus* [18], which could partially explain the results from the new method. It is a common finding that NAS isolated from TA are more likely to produce antagonist substances against other bacteria [15,29]. Moreover, protection against *S. aureus* by the *S. simulans* species has previously been suggested [18,30], although most of the studies have reported more pronounced inhibitory effects by *S. chromogenes* [15,23,24,29] and a modest effect by *S. epidermidis* isolates. The effect observed for the latter species is most probably because these species are usually transferred from human skin to cows during the milking process [37,38] and their virulence properties are limited [39]. Nevertheless, considering the significant interaction observed, we showed that the growth-inhibitory effect of isolates from the different NAS habitats was not equal across species.

Lastly, the contamination rate observed by the new method should be considered a limitation of this study. The new technique is more elaborate than the semiquantitative method and needs sufficient experience to be used successfully. The semiquantitative method would be recommended for first screening a large group of NAS, whereas the novel assay is applicable to measure in vitro growth inhibition of major mastitis bacterial pathogens (including MRSA) by bovine NAS in more detail.

This method contributes to the knowledge on the interaction of bovine NAS and other bacterial pathogens, gauging for new venues for preventive (e.g., new probiotics) or therapeutic measures (e.g., identification of new antibiotic compounds, doses, and spectrum of inhibition) for bovine mastitis. Further studies applying the new assay should include more NAS species originating from more diverse habitats to reveal greater diversity in NAS inhibition. As well, we believe the method will also be useful in studying the interaction between NAS and other mastitis pathogens. 

## 4. Materials and Methods

### 4.1. General Study Design

Firstly, a novel assay was developed to quantitatively measure whether different NAS isolates (*S. chromogenes* (*n* = 3), *S. simulans* (*n* = 3)) could inhibit the growth of bovine major mastitis bacterial pathogens (*S. aureus* (*n* = 10), *E. coli* (*n* = 10), and *S. uberis* (*n* = 10)). Subsequently, the results were compared with the semiquantitative in vitro growth inhibition method described by De Vliegher et al. [15]. 

Secondly, the novel assay was used to quantify the capacity of 14 other bovine NAS (*S. chromogenes* (*n* = 6), *S. epidermidis* (*n* = 4), and *S. simulans* (*n* = 4)), a subset of the isolates used by Toledo-Silva et al. [18,19], to inhibit the growth of both bovine MSSA (*n* = 5) and MRSA isolates from milk (*n* = 5) in vitro. In addition, it was checked whether the growth inhibition was related to specific NAS (species, origin (milk *versus* teat apex) and ability to repress the *agr* system of *S. aureus*) and *S. aureus* traits (antimicrobial susceptibility).

### 4.2. Description of the Novel Quantitative Growth Inhibition Assay

#### 4.2.1. Bacterial Isolates and Growth Conditions

Three *S. chromogenes* and three *S. simulans* isolates were selected for this experiment based on phenotypical differences, including “*S. chromogenes* IM”, an udder-adapted strain that was isolated from a cow with a persistent intramammary infection [10] and “*S. chromogenes* TA”, a well-described inhibitor which originates from a healthy heifer’s teat apex [15]. The other *S. chromogenes* and all 3 *S. simulans* isolates were isolated from milk and caused a persistent (EL12, EL14, and EL29) or transient (EL38) IMI [7,10].

In addition, different field major mastitis bacterial pathogen isolates recovered from milk samples were selected from our repository (*S. aureus* (*n* = 10), *E. coli* (*n* = 10), and *S. uberis* (*n* = 10)). These isolates originated from different dairy herds in Flanders (Belgium) and were isolated between 2012 and 2017. 

The species-level identification of all isolates was verified with matrix-assisted laser desorption/ionization time-of-flight mass spectrometry (MALDI-ToF MS) [40] using the commercially available Bruker database and our updated NAS library [41,42,43].

After species identification, a pure culture of all isolates was grown (NAS and major pathogens) to establish a growth curve. One single colony was picked up with a sterile inoculation loop and grown in a sterile flat-bottom 96-well plate (Thermo Scientific, Waltham, MA, USA) under aerobic conditions at 37 °C in brain-heart infusion (BHI; Oxoid, Hampshire, UK) inside a spectrophotometer (Multiskan GO, Thermo Scientific, Waltham, MA, USA). The late logarithmic growth phase was determined for each NAS and major mastitis bacterial pathogen isolate by measuring the optical density of the bacterial solution every 30 min for 20 h.

Next, bacteria were grown to the late logarithmic growth in BHI, and sterile glycerol was added to the bacterial culture to obtain a 30% (*v/v*) glycerol stock that was stored at −80 °C. The viability and concentration of each bacterial glycerol stock was tested in triplicate. One-hundred microliter of the bacterial stock was incubated in 5 mL sterile BHI at 37 °C under aerobic conditions until bacteria reached the late logarithmic growth phase. Subsequently, 50 µL of the latter bacterial solution was enriched with 5 mL fresh sterile BHI medium and aerobically incubated for 18 h at 37 °C. Overnight cultures (18 h) were washed twice using sterile phosphate buffered saline solution (PBS) (Thermo Scientific, Waltham, MA, USA) and centrifuged at 3220× *g* for 10 min. Next, serial dilutions were plated on tryptic soy agar (TSA; Oxoid, Basingstoke, UK) and the plates were aerobically incubated for 24 h at 37 °C before the number of colony-forming units (CFU)/mL were calculated.

#### 4.2.2. Novel Quantitative Growth Inhibition Assay

**Figure 3:**
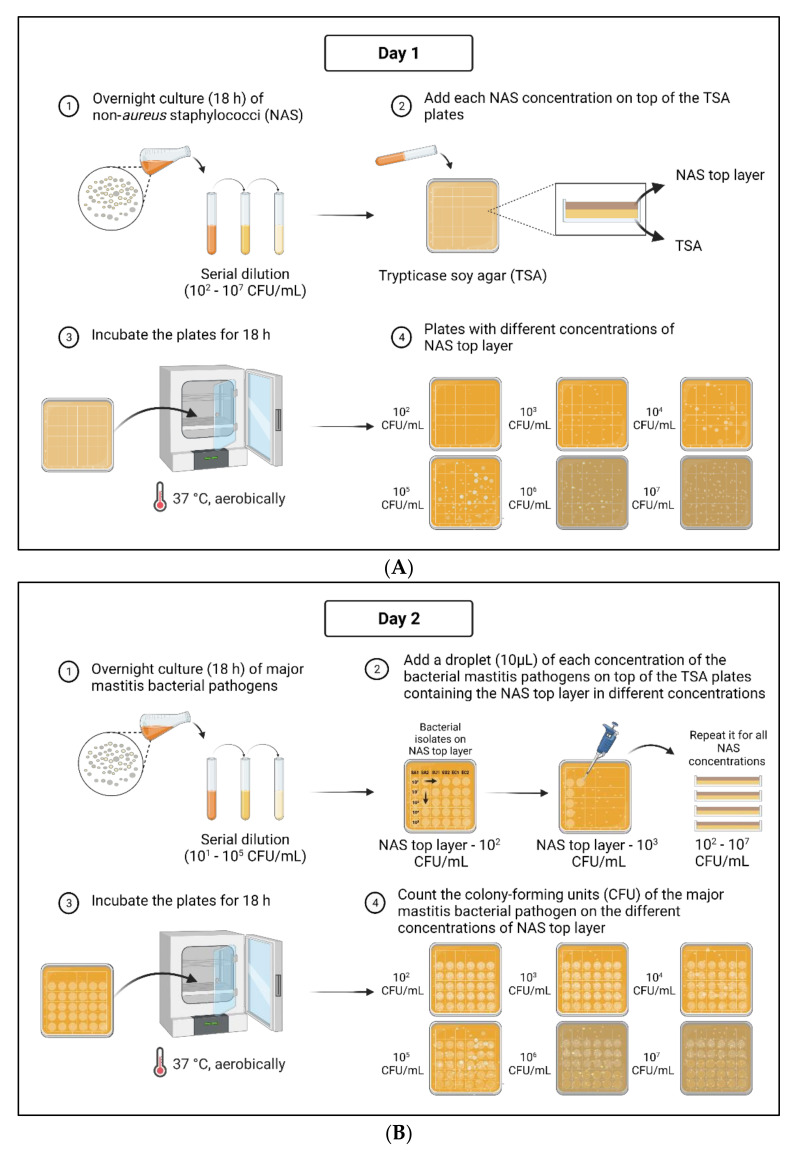
Schematic overview of the novel quantitative method to evaluate growth-inhibitory activity of non-*aureus* staphylococci (NAS) of major mastitis bacterial pathogens. On day one, top layers of NAS with different concentrations are prepared (**A**) and on the second day the major mastitis bacterial pathogens are added on top of the plates containing the different NAS at different concentrations (**B**).

A schematic overview of the methodology is described in the Figure 3.

Briefly, cultures of the *S. aureus, E. coli*, and *S. uberis* isolates were incubated for 18 h (as described for the NAS isolates) and a top layer with a NAS isolate was created as follows. On day 1 (Figure 3A), the NAS cultures, previously incubated for 18 h, were washed and centrifuged twice (as described above) and diluted with sterile PBS to obtain a concentration of 108 CFU/mL. Subsequently, a 1:10 dilution series was created in sterile PBS. One hundred microliters of six different dilutions (10^3^, 10^4^, 10^5^, 10^6^, 10^7^, and 10^8^ CFU/mL) were added to 10 mL sterile 2% TSA solution in a Falcon tube that was kept warm (50 °C) immediately after autoclaving to avoid solidification. This resulted in a final NAS concentration of 10^2^, 10^3^, 10^4^, 10^5^, 10^6^, and 10^7^ CFU in a 10 mL top layer. The Falcon tube was vortexed and poured on a sterile, pre-heated (37 °C) TSA plate. The solution was distributed equally, and the plate was left to solidify. As a control, 100 µL sterile PBS was added to 10 mL warm TSA solution and poured on a sterile TSA plate and left to solidify. The abovementioned steps were repeated five times for each concentration of each NAS isolate and for the controls. After solidification, all plates were turned upside down and aerobically incubated for 18 h at 37 °C. Serial dilutions of each concentration of each NAS isolate were plated on TSA to confirm the dose.

On day 2 (Figure 3B), the plates containing the NAS isolates were checked for growth and contamination. Subsequently, cultures of the *S. aureus*, *E. coli*, and *S. uberis* isolates, previously incubated for 18 h, were washed with sterile PBS as described above. The bacterial solutions were first diluted in sterile PBS to obtain a concentration of 10^7^ CFU/mL, and subsequently serial dilutions were made in sterile PBS. One droplet (10 µL) of five different concentrations (10^5^, 10^4^, 10^3^, 10^2^, and 10^1^ CFU/mL) of five major mastitis bacterial pathogen isolates was put on a plate of each concentration of each NAS isolate. Droplets of each concentration of each major mastitis bacterial pathogen isolate were also inoculated on a TSA plate with a top layer containing 100 µL sterile PBS. These plates served as negative controls and were also used to confirm the concentration of each major mastitis bacterial pathogen isolate. The abovementioned steps were repeated for all major mastitis bacterial pathogen isolates. All plates were aerobically incubated for 18 h at 37 °C.

After 18 h of incubation, the number of CFU/mL of each major mastitis bacterial pathogen isolate was calculated by counting the CFU/droplet in the dilution that contained approximately 3 to 50 CFU/droplet.

#### 4.2.3. Semiquantitative Growth Inhibition Assay

In order to compare the results of the new quantitative assay with the previously used semiquantitative in vitro growth inhibition method, we performed this assay as described before [15] for all NAS isolates in combination with one *S. aureus* isolate (in duplicate), one *E. coli* isolate (in triplicate), and one *S. uberis* isolate (in triplicate).

### 4.3. Application of the Novel Quantitative Growth Inhibition Assay

#### 4.3.1. Bacterial Isolates and Origin

Fourteen NAS isolates were obtained from our repository and were selected for this experiment based on their specific traits (Table 2), including the “*S. chromogenes* TA” and “*S. chromogenes* IM” (see before). The other NAS isolates represent the three most prevalent species in milk samples (*S. chromogenes* (*n* = 3), *S. epidermidis* (*n* = 3), and *S. simulans* (*n* = 3)) and on teat apices of dairy cows and heifers (*S. chromogenes* (*n* = 1), *S. epidermidis* (*n* = 1), and *S. simulans* (*n* = 1)) [7,44].

The 10 *S. aureus* isolates (five MRSA and five MSSA) were isolated in Belgium and Norway from quarter milk samples of cows with clinical or subclinical mastitis [22]. 

Species identification (MALDI-ToF) and growth of all isolates were performed under the abovementioned conditions (Bacterial isolates and growth conditions).

#### 4.3.2. Application of the Assay 

All assays were performed under identical conditions as described before (novel assay) using the new subset of NAS isolates (Table 2). 

### 4.4. Statistical Analyses

#### 4.4.1. Description of the Novel Quantitative Growth Inhibition Assay

First, a non-parametric Kruskal–Wallis test was run to compare the capability of the novel assay to detect the growth inhibition of *S. aureus*, *E. coli,* and *S. uberis* in vitro by NAS, with the semiquantitative method.

Second, two additional non-parametric Kruskal–Wallis tests were run to compare growth inhibition of *S. aureus, E. coli,* and *S. uberis* by the six different NAS isolates (EL12, EL14, EL29, EL38, TA, and IM) (1), and between NAS with the same capacity to inhibit growth of *S. aureus, E. coli*, and *S. uberis*, respectively, according to the semiquantitative assay (2) (version 26, SPSS, Chicago, IL, USA). Significance was assessed at *p* < 0.05.

#### 4.4.2. Application of the Novel Quantitative Growth Inhibition Assay 

Firstly, a Cox proportional hazard model was fit with the log_10_ NAS concentration (CFU/mL) at which the growth of *S. aureus* was inhibited as the time to event (i.e., *S. aureus* concentration equals 0) to compare *S. aureus* growth inhibition as detected via the novel assay and the *S. aureus* growth inhibition detected via the semiquantitative assay (total, partial, or no growth inhibition; [17]) (version 9.4, SAS Institute Inc., PROC MIXED).

Secondly, four similar separate Cox proportional hazard models were fit to understand whether (1) different NAS species (*S. chromogenes, S. epidermidis,* and *S. simulans*), (2) different habitats (milk or teat apices), or (3) differences in the ability of NAS to affect the *agr* system of *S. aureus* (no effect, slight effect, moderate effect, or severe effect; [17]) explained variation in growth inhibition of the *S. aureus* isolates. The final model was fit to evaluate whether the capability of NAS to inhibit growth of *S. aureus* according to the novel assay differed between MRSA and MSSA (4). 

Finally, a multivariable Cox proportional hazard model was fit with the log_10_ NAS concentration (CFU/mL) at which the growth of *S. aureus* was inhibited as the time to event (i.e., *S. aureus* concentration equals 0), including the different NAS species, the different habitats, and the interaction between both variables as independent categorical variables, to check whether the habitat effect was similar over the three species.

In all models, observations were coded as right censored when *S. aureus* isolates were still growing at the highest tested NAS concentration. The results were expressed as hazard ratios and their 95% confidence intervals. When the confidence interval included 1, then the hazard ratio was deemed non-significant. For all models, Kaplan–Meier survival curves were plotted.

## 5. Conclusions

In conclusion, we showed that the new quantitative in vitro growth inhibition method is appropriate to evaluate and extensively quantify the potential protective effects of bovine NAS against major mastitis bacterial pathogens. The in vitro growth inhibition of *S. aureus, S. uberis,* and even *E. coli* by NAS isolates was variable, a finding that was not evident from the semiquantitative in vitro growth inhibition method. Furthermore, lower concentrations than those in the semiquantitative method were required to reveal *S. aureus* growth inhibition (including MRSA isolates) by *S. simulans*, NAS originating from TA, and NAS with a strong or moderate capacity to repress the *S. aureus agr* system. Our findings contribute to the knowledge of interactions between bovine NAS and major mastitis bacterial pathogens. The new method is a promising tool to display intra- and inter-species differences between NAS in inhibitory activity.

## Figures and Tables

**Table 1:** In vitro growth inhibition of major mastitis bacterial pathogens by six NAS isolates using the semiquantitative method previously described by De Vliegher et al. [15].

NAS Isolate	*S. aureus*				*E. coli*				*S. uberis*			
	T ^4^ (mm)	P ^5^ (mm)	N ^6^ (mm)	C ^7^	T (mm)	P (mm)	N (mm)	C	T (mm)	P (mm)	N (mm)	C
*S. chromogenes (S. c.)*												
*S. c.* IM ^1^	0	12	23	p p	0	2	33	n n p	0	3	32	n p t
*S. c.* TA ^2^	7	10	18	t t	0	2	33	p n p	0	3	32	n p p
*S. c.* EL14 ^1^	0	15	20	p p	0	0	35	n p p	0	1	34	p p p
*S. simulans (S. s.)*												
*S. s.* EL12 ^3^	0	15	20	p p	0	0	35	n p n	0	3	32	p p p
*S. s.* EL29 ^1^	0	15	20	p p	0	0	35	p n n	0	2	33	n p p
*S. s.* EL38 ^1^	0	15	20	p p	0	0	35	n p n	0	1	34	p p p

^1^ Supré et al. [10]; ^2^ De Vliegher et al. [15]; ^3^ Piessens et al. [7]; ^4^ T: Zone of total growth inhibition (mm); ^5^ P: Zone of partial growth inhibition (mm); ^6^ N: Zone of no growth inhibition (mm); ^7^ C: Central-streak zone of NAS isolate (always 5 mm wide) (NAS isolate at the backside of the agar; t: total growth inhibition; p: partial growth inhibition; n: no growth inhibition).

**Table 2:** Origin and traits of bovine *S. chromogenes* (*S. c.*), *S. epidermidis* (*S. e.*), and *S. simulans* (*S. s.*) isolates.

Species and Isolates	Origin (cow)	*S. aureus* Growth Inhibition ^3^	*agr* Repression ^3^
*S. chromogenes (S. c.)*			
*S. c. 1—*“IM” ^1^	Milk	Partial	Weak
*S. c. 2*	Milk	Partial	Weak
*S. c. 3*	Milk	Partial	No
*S. c. 4*	Milk	Partial	Moderate
*S. c. 5—*“TA” ^2^	Teat apex	Total	Strong
*S. c. 6*	Teat apex	Partial	Weak
*S. epidermidis (S. e.)*			
*S. e. 1*	Milk	Partial	No
*S. e. 2*	Milk	No	No
*S. e. 3*	Milk	No	No
*S. e. 4*	Teat apex	Partial	No
*S. simulans (S. s.)*			
*S. s. 1*	Milk	Partial	Moderate
*S. s. 2*	Milk	Partial	Weak
*S. s. 3*	Milk	Partial	Strong
*S. s. 4*	Teat apex	Total	Moderate

¹ Obtained from Supré et al. [10]; ^2^ Obtained from De Vliegher et al. [15]; ^3^ Results from Toledo-Silva et al. [18], obtained by the semiquantitative method [15].
